# Genetic analyses of birthweight and cardiovascular disease

**DOI:** 10.1093/eurheartj/ehae510

**Published:** 2024-10-03

**Authors:** Maddalena Ardissino, Stephen Burgess, Fu Siong Ng

**Affiliations:** 1 National Heart and Lung Institute, Imperial College London, Du Cane Rd, London W12 0NN, UK; 2 Cardiovascular Epidemiology Unit, Department of Public Health and Primary Care, University of Cambridge, UK; 3 Medical Research Council Biostatistics Unit, University of Cambridge, UK

**This commentary refers to ‘Birth weight influences cardiac structure, function, and disease risk: evidence of a causal association’, by M. Ardissino *et al*., https://doi.org/10.1093/eurheartj/ehad631 and the discussion piece ‘Is lower birthweight truly causal for increased cardiovascular risk?’, by N. Warrington *et al*., https://doi.org/10.1093/eurheartj/ehae509.**

We thank Warrington *et al*.^1^ for their interest and discussion regarding our recent study on the relevance of birthweight in the risk of cardiovascular disease^2^ using Mendelian randomization (MR). When interpreting MR findings in this context, three key issues arise: the validity of the statistical analyses, the interpretation of the findings, and the biological relevance of the results.

With regard to the statistical analyses, we concur on the importance and methodological challenges of controlling for maternal genetic influences in MR analyses. While we utilized variants adjusted for, or isolated from, maternal genetic effects (using multiple different methods that we describe in the paper), we recognize that these adjustment methods are not flawless, which raises the possibility of residual horizontal pleiotropy. While we acknowledge that this is a potential explanation for the discrepancy with a previous analysis of mother-offspring pairs, the lack of association in this previous study could also in large part be explained by the limited cohort size and further reduction in power of the analysis after adjustment for correlation between maternal and offspring genotypes.^3^ In our study, we employed multiple methods to detect any evidence of pleiotropy, and none indicated any concerns. However, acknowledging the inherent limitations of these methods, we are openly aware that its absence cannot be guaranteed and highlight this as an important limitation in the paper.

As for the interpretation of the findings, we agree that results of MR analyses should not be taken unquestioningly as proof of a causal role, but rather as pieces of evidence to support or refute one, when taken together with other knowledge on the subject. Objectively, the findings of our analysis at the very least provide a piece of the puzzle in support of a role of birthweight in cardiovascular disease risk, which should be taken together with the totality of evidence, including the studies referred to by Warrington and colleagues.^3^

Regarding the biological relevance of the results, Warrington and colleagues argue that the findings lack practical relevance. We disagree. From a public health perspective, large-scale efforts to mitigate the health burden of low birthweight and growth restriction through the intrauterine environment are important for reasons beyond later life cardiovascular disease, such as the reduction of stillbirth and neonatal morbidity. Whether effects on cardiovascular disease operate via intrauterine environment directly or via birthweight is important from an aetiological perspective, to understand whether it is worth targeting cardiovascular prevention strategies at low birthweight individuals. However, we note that our analyses do not preclude an effect of intrauterine environment that operates via birthweight, hence intrauterine environment is an important modifiable risk factor for cardiovascular disease even if it only affects disease risk indirectly. Overall, we therefore believe that our result remains an important association with potential practical implications that is worthy of further exploration and understanding.
